# Experiences of support for people who access voluntary, community and social enterprise (VCSE) organisations for self-harm: a qualitative study with stakeholder feedback

**DOI:** 10.1186/s12889-024-18455-4

**Published:** 2024-04-16

**Authors:** Joe Hulin, Vyv Huddy, Phillip Oliver, Jack Marshall, Aarti Mohindra, Brigitte Delaney, Caroline Mitchell

**Affiliations:** 1 Sheffield Centre for Health and Related Research (SCHARR), School of Medicine and Population Health, University of Sheffield, Sheffield, UK; 2 Department of Psychology, University of Sheffield, Sheffield, UK; 3 Primary Medical Care Research, School of Medicine and Population Health, University of Sheffield, Sheffield, UK

**Keywords:** Self-harm, Qualitative, Third sector, Peer support, Self-injury, Mental health

## Abstract

**Background:**

Prevalence of self-harm In England is rising, however contact with statutory services remains relatively low. There is growing recognition of the potential role voluntary, community and social enterprise sector (VCSE) organisations have in the provision of self-harm support. We aimed to explore individuals’ experiences of using these services and the barriers and facilitators to accessing support.

**Methods:**

Qualitative, online interviews with 23 adults (18+) who have accessed support from VCSE organisations for self-harm in the Yorkshire and the Humber region were undertaken. Interviews were audio recorded and transcribed verbatim. Thematic analysis was undertaken using NVivo software.

**Results:**

Participants described how a lack of service flexibility and the perception that their individual needs were not being heard often made them less likely to engage with both statutory and VCSE organisations. The complexity of care pathways made it difficult for them to access appropriate support when required, as did a lack of awareness of the types of support available. Participants described how engagement was improved by services that fostered a sense of community. The delivery of peer support played a key role in creating this sense of belonging. Education and workplace settings were also viewed as key sources of support for individuals, with a lack of mental health literacy acting as a barrier to access in these environments.

**Conclusions:**

VCSE organisations can play a crucial role in the provision of support for self-harm, however, pathways into these services remain complex and links between statutory and non-statutory services need to be strengthened. The provision of peer support is viewed as a crucial component of effective support in VCSE organisations. Further supervision and training should be offered to those providing peer support to ensure that their own mental health is protected.

## Background

Self-harm is defined by the National Institute for Health and Care Excellence (NICE) in 2023 as ‘any act of self-poisoning or self-injury…. irrespective of motivation’ and is a major public health concern [1]. Self-harm has been identified as a key indicator for suicide risk; it is the strongest risk factor for suicide in children and young people and approximately 50% of individuals who take their own life have a history of self-harm [2, 3]. In this context, self-harm has been highlighted as one of the 7 key areas requiring action as recommended by Public Health England in 2017 [4].

Research drawing on the British Psychiatric Morbidity Survey has found the prevalence of self-harm has increased, particularly in younger age groups, and in females [5]. This study reported that prevalence increased from 6.5% in 2000 to 19.7% in 2014, in females aged 16–24 years [5]. The same research has shown that the number of people seeking medical or psychological support from statutory services appears to be stagnant, and a majority do not seek formal help [5]. Barriers to accessing support across all age groups include short consultation times, stigma related to mental health issues and the idea of ‘attention seeking’, confidentiality concerns and GP consultation skills [6–8]. Older adults who self-harm also perceive that their physical health problems can feel prioritised over their mental health struggles due to time constraints in primary care [8]. A further barrier appears to be the perception that GP’s lack skills in working with mental health issues and that primary care professionals only view physical health problems as important [6, 7].

Informal help can be defined as support from family, friends, peers, religious leaders or other non-health professions including non-statutory or voluntary sector organisations. Research on support for broad categories of mental health difficulties has found that this type of support is very widely used [9]. For example, data collected in 2008–2010, showed that 60% of 386 UK adults with common mental health disorders seeked informal help [9]. Despite this, little is known about the experiences of people who access such support for self-harm. A review of peer support groups for self-harm, published in 2022, found that such groups can have a therapeutic role, with social and emotional benefits [10]. However, the studies included in this review were mostly focused on on-line peer support and did not position the findings in the context of general experience of accessing support or why informal, VCSE organisations might be used.

Our study aimed to qualitatively explore the experiences of those accessing VCSE support in a group of adults with lived experience of self-harm to determine the barriers and facilitators to accessing and engaging with services.

## Methods

### Study design

A qualitative study was undertaken using semi-structured interviews with a purposive sample of individuals with lived experience of self-harm. A lived experience advisory group (LEAG) met regularly with researchers and contributed to all stages of the research process.

### Participants and recruitment

We recruited individuals via 72 VCSE organisations providing support for individuals who self-harm in the Yorkshire and the Humber (YH) region. VCSE organisations across the region were contacted via phone or email by a member of the study team and asked to advertise the study in their services and via social media. The advert was co-created with our LEAG and participants received a £10 gift voucher for taking part.

Inclusion criteria for participants were:

- aged 18 years or over.
- self-identified as having lived experience of self-harm as defined by NICE [1].
- living in the YH region.

Participants self-selected to take part by contacting a member of the study team via email or telephone. Eligible participants were provided with an information sheet and an online consent form. All participants were given the opportunity to ask questions about the research via email or through an informal discussion with a member of the research team. Participants were informed that their decision to participate would not impact on the support they were receiving from any organisation and that they could withdraw from the research at any stage. Consenting participants also completed a sociodemographic form which collected data on age, gender, ethnicity, education, employment, and location.

Purposive sampling was attempted when under-recruiting by key demographic characteristics. This included individuals from ethnic minority groups, males and adults aged over 55 years. Interviews continued until data saturation was achieved in a maximum variety sample.

### Procedure

Semi-structured, one to one interviews using a topic guide were undertaken online or via telephone by a member of the interdisciplinary research team (BD, JH, JM or AM) from October 2021 to April 2022. The content of the topic guide was informed from existing literature, policy documents and discussions with our LEAG. For the online interviews, the audio-visual and chat box messages were recorded using the in-built systems on Google Meet and Blackboard Collaborate Ultra. Visual recordings were converted to an mp3 format and the original mp4 recording deleted. Audio from telephone interviews was recorded using dedicated encrypted recording devices. All interviews were transcribed verbatim and interview transcripts were anonymised in order to protect the identity of participants (e.g. names of people, locations or services were redacted). All audio files were deleted following transcription and data was stored on a secure drive in accordace with University policy.

### Data analysis

Thematic analysis, using NVivo software, was undertaken in accordance with Braun and Clarke’s framework [11]. This framework includes the following six phases: familiarisation, generating initial codes, searching for themes, reviewing themes, defining and naming themes and producing the report. In accordance with this framework, researchers (JM, AM and JH) read and re-read transcripts to familiarise themselves with the data and initial notes on impressions of the participants’ accounts were recorded. Researchers (JM, AM and JH) coded the transcripts separately before sorting codes into broader candidate themes and sub themes. These preliminary themes were subject to critical interpretive challenge during regular analysis workshops with the whole research team to achieve a coherent and consistent account of the data. The themes were also presented to the LEAG to be defined further and named. The final findings were presented for further feedback in an online workshop to the members of our LEAG and 70 key stakeholders, including mental health practitioners, public health practitioners, academics, and individuals working in VCSE organisations.

Involvement of the LEAG throughout the research study design allowed us to address the four key principles of validity of qualitative research [12]. For example, through presenting preliminary themes to individuals with lived experience of accessing support for self-harm, we were able to ensure we were not imposing our pre-conceived categories onto the data and consequently demonstrating “sensitivity to context” [12]. The principle of “commitment and rigour” [12] was also achieved through the iterative phases of data analysis, which enabled prolonged and in-depth engagement with the data. Demonstrating transparency is also highlighted as an indicator of quality in qualitative research [12], which was addressed by ensuring the themes were grounded in examples and quotes from participants. Finally, impact and importance were addressed by ensuring that key stakeholders were involved in the research from the design phase so that the research question would be of practical usefulness for organisations providing support. The online presentation of findings to members of our LEAG and other key stakeholders, also allowed the team to gain additional perspectives on the themes uncovered and use these stakeholder perspectives to develop implications for public health policy and practice.

## Results

Twenty three interviews were undertaken. The majority of participants were female (*n* = 17) and white British (*n* = 19) (see Table 1). All participants were aged over 18 years, with the majority of inividuals (*n* = 8) aged 26–35. Five participants were aged 56 and over. Twelve of the study population lived in areas falling within the 5th or higher index of multiple deprivation (IMD) decline. In terms of employment, 14 participants were employed and 5 were unemployed. One individual was a University student, one was retired and one participant preferred not to answer the question.

Whilst the focus of our research was on accessing support from VCSE organisations, general experiences of seeking support from all services were captured, allowing us to explore whether barriers and faciltators to access and engagement differed across statutory and non-statutory services. Four key themes were identified from the data: appropriate and timely support, fostering a sense of community, awareness of support and employment and education. Table 2 summarises these main themes and explanatory sub-themes. The theme of timely and appropriate support included two main sub themes relating to the need for person-centered care and additional support in navigating complex care pathways. What participants defined as appropriate support was also partially addressed in the second theme, which highlighted the need for services to foster a sense of community. In this theme participants highlighted the importance of shared experiences, across both in-person and online resources. The third theme covered the perception that there was a lack of information available on existing support across both statutory and non-statutory services. Finally, the importance of employment and education settings in providing support for self-harm was also emphasised.

### Appropriate and timely support

This theme captured how individuals perceived a lack of person-centred services for self-harm, which provided support in a timely manner. Participants described the need for additional support in navigating complex care pathways, which included multiple referrals and lengthy delays in accessing support.

#### Tailored services

Participants described the importance of services being tailored to meet individual needs and how a lack of options made them less likely to engage with services. For example, one individual described how they were hesitant to access support from one VCSE organisation, because they felt their preferences for one-to-one support were not being heard by healthcare professionals:

I said I’ll think about it on the phone but when I thought about it I knew deep down I really wanted one to one. It kind of upset me a bit. I’m not sure why, maybe because I thought they were just going to get rid of me or something, I don’t know, but yeah that kind of threw me off. If I’d have wanted group therapy, I would have said it at the beginning.

P20: White British Female, aged 26–35 years

An inability or unwillingness for services to tailor approaches to meet the needs of individuals with neurodevelopmental conditions, such as autism, was also highlighted:

It’s one size fits all, we don’t want to have any specialised services, we don’t want that because it’s too much trouble, too much expense.

P16: White British Female, aged 56–65 years

Practical issues, such as the time in which sessions were being delivered, was another potential barrier. For example, one participant described how a lack of service flexibility made it difficult to access support from some VCSE organisations, particularly for those in full time employment:

groups are like three and a half hours a week with everything and I work full time, so it was kind of, like, you know, it’s stressful enough working without trying to suddenly find more time as well.

P3: White British Female, aged 26–35 years

Accessibility to third sector support was also dependent on the location in which you live, with one individual describing how living in a more rural environment, with fewer transport links, meant appropriate support was not always available:

place X is really, really low in third sector availability and transport. That’s a particular issue for me; I live in a little village. Accessibility is a huge problem.

P11: White British Female, aged 56–65 years

#### Navigating complex pathways

The complexity of care pathways also limited access to services. One individual described how “you’ve got to be the right sort of mad at the right time” (P11: White British Female, aged 56–65 years) to be able to receive appropriate support. Both statutory and non-statutory services were often viewed as fragmented, and some participants highlighted a perceived lack of support in helping navigate these care systems, leading to feelings of isolation:

So then I was a little bit sort of taken aback then, it’s like ‘well where do you want to go from here’ and I’m like ‘well I don’t really know, I was hoping you’d kind of help me’, but then, no, so then I was just kind of on my own again.

P3: White British Female, aged 26–35 years

One participant felt that better communication between services could help staff or volunteers guide individuals through these care pathways more effectively, enabling smoother care transitions. The importance of building rapport through continuity of care was also viewed as a key component of care:

I think it’s better communication between the teams… like my facilitator in DBT (Dialectical Behaviour Therapy) is also on the crisis team so you sort of build, yeah you sort of build a rapport with that person.

P13: White British Female, aged 26–35 years

Navigating pathways were also made more difficult as a result of waiting times following initial referrals:

So he was like ‘go away and try and some talking therapies and come back’, so I spent two years trying to access talking therapies, kept getting taken off the waiting list without being told, and then it’s probably, I’d say January this year, I went back to the GP and I was like ‘look, I can’t do anything about this, it’s worse’, so then she prescribed me some medication for my anxiety and then I was like ‘OK and what about anything else’ and then she was like ‘well you’ll just have to self-refer and you’ll just have to find something to keep you going, I can’t really refer you on or do anything about that, you’ll have to go and find somewhere to refer yourself to or somewhere to access support’. So I referred myself back to the Mental Welling being Service. That was in January, they said it would be an eight month wait to bet CBT (Cognitive Behavioural Therapy)– never heard back from them since.

P5: White British Female, aged 18–25 years

The time required to access support was seen as a particular problem for some individuals, who felt that they needed more urgent responses from services to help with their self-harm. It was suggested that more immediate support was often facilitated through drop-in services:

I struggle with the waiting times, because it’s like by the time it comes around sometimes I might be OK and not be able to explain it properly, so with the drop-ins (via the University health service) you could literally just go when you wanted and you had the counselling service, which you did have to wait for a bit, but they still had this like wellbeing person that you drop in with and stuff.

P3: White British Female, aged 26–35 years

### Fostering a sense of community

Individuals were more likely to engage with support in settings which fostered a sense of belonging. Some individuals highlighted how being a valued member of a community aided their recovery:

People don’t recover in our institutions, they recover in community. Once you’ve been accepted within a community and welcomed within a community and seen a value asset within a community and that you’ve got a purpose of you’ve got something to give.

P1: White and Black Caribbean Male, aged 46–55 years

#### Shared experiences

Environments which fostered a sense of community were often established through the inclusion of peer support, with individuals noting how they were more comfortable seeking support from those with shared experiences:

Group X (VCSE organisation), which is a peer support group which really helps me and it allows me to talk about my experiences without being ashamed of it, because there’s other people with similar experiences. So being part of Group X makes me feel as though I belong somewhere.

P1: White and Black Caribbean Male, aged 46–55 years

One individual also discussed how being able to support others was also viewed as having a positive impact on their own wellbeing and mental health:

I find it quite helpful because it’s like I try to be the person that I needed when I was younger, that person who is safe to talk to who will fight for the underdog.

P17: White British Female, aged 26–35 years

However, the potential burden this could have on those providing peer support in VCSE settings was also acknowledged, with one individual highlighting the fact that “you can’t hang everything on people who are struggling themselves”.

P11: White British Female, aged 56–65 years

#### Online communities

Participants also highlighted how a sense of community or belonging could often be created through engagement with others online:

*“It’s like an online Big White Wall so with like post-it notes and different little forums where people can talk about stuff. And the main message is like “You’re not alone” which is a really important thing with self-harm; you do feel like “Oh my God, what’s going on with me? I’ve never experienced this before”.*

P22: White British Female, aged 18–25 years

Below one participant describes how coining of new phrases regarding a perceived lack of need for support helped them recognise that others share similar experiences:

She’s come up with this thing basically called ‘Baby Cut Syndrome’…. so it’s kind of, like, the whole baby cut, you think yours aren’t as deep as someone else sort of thing, so that’s quite a useful sort of term that kind of I feel I wouldn’t have known other people had until that came up and it suddenly sort of clicked and made sense… it’s just kind of a feeling that you think you always have on your own but then knowing that other people have that feeling too and there’s a name for it, that she’s come up with, is quite helpful, quite validating in a way.

P3: White British Female, aged 26–35 years

However, it was recognised that shared experiences of mental health on social media can negatively impact individuals by acting as a “trigger” for their self-harm (P3: White British Female, aged 26–35 years).

### Awareness of support

Some individuals felt that access to support was hindered by a lack of awareness of available services and by not being provided with a clear understanding of the support they would likely receive.

#### Knowledge of service availability

Participants described how they felt existing services were not clearly communicating the support available:

I think had I known where to go sooner and had it been talked about more, I might have stopped self-harming sooner or sought out services sooner.

P22: White British Female, aged 18–25 years

One individual also described how they had a perception that self-harm would not be addressed in primary care:

…it feels like too small of an issue to go to a GP with, because I feel like depression is a big enough issue, any other mental illness is a big enough issue, but self-harm is like this tiny little subcategory where it’s like, you know, yeah, so I think I was like oh I’ll manage it.

P7: Indian female, aged 18–25 years

This lack of awareness was also reported to be mirrored by those involved in delivering support:

…in the conversation with the receptionist I just said mental health and then she started panicking being ‘oh we’re not equipped to deal with this, we can’t…’, like, I kept it very, very general and then she was like ‘we’re not equipped to deal with this, you couldn’t be calling us, you should be calling someone else, blah blah blah’ and I was like, at the time, I was like ‘well it’s only anxiety, I’m pretty sure a GP can deal with that, but then because she had such a panic reaction, I was like well I’m not going to bother going about self-harm or anything like that, because she made it sound like they’re not equipped to deal with anything that could be classed as a bit more serious.

P5: White British Female, aged 26–35 years

#### Lack of clarity

The lack of information on what services entailed also had a significant impact on engagement with support. One individual described how they did not know what to expect from service and this made them more reluctant to access care:

…a bit more clarity would be good, because I got told I was going to this core psychology and that I had this meeting, but I didn’t know what it was, how long it were going to be for, who it was with and it ended up being with a psychologist rather than a…like a counsellor or summat, I didn’t know whether it was going to be a weekly counselling thing or what or whether I’d meet her once and then I’d have to wait for something else….

P3: White British Female, aged 26–35 years

### Education & employment

Participants described the importance of creating supportive environments in education and work settings for people who self harm. The role employment can play in creating a sense of meaning or purpose, which can help facilitate personal recovery, was also highlighted.

#### Creating supportive environments

Employment settings were highlighted as sources of support for some individuals. However, a lack of mental health literacy among employees and available resources was noted as a particular barrier to accessing support:

…my direct manager isn’t the most understanding person of mental health…she’s not able to recognise signs, symptoms of like people who are in crisis or whatever, you know, but her manager is a little bit more, so I’ve gone and spoken to her about stuff, and yeah so she recommended that I go to see the OTs (Occupational Therapists) and I’ve tried contacting them but they’ve never come back to me…even when you ask for that help they’re not always there.

P17: White British Female, aged 26–35 years

Support in education settings was also seen as crucial for some individuals, especially when students felt academic pressure was having a negative impact on their mental health:

my tutor was brilliant as well and they were just really helpful because they didn’t– they let me have like extensions, they didn’t put pressure on me in terms of my work and things, they were just brilliant, can’t say a bad word about uni really.

P3: White British Female, aged 26–35 years

#### Empowerment and self esteem

It was also noted how employment opportunities could play a role in improving individuals mental health and wellbeing and reducing experience of self-harm:

I’d been doing volunteering all along but then when I started to get into paid work, that was really massive for me in terms of like self esteem, self worth, like structure, routine, all of that kind of thing.

P18: White British Female, aged 36–45 years

This sub theme, is closely related to the sub-theme of “Shared Experiences”, in which the positive impact on mental wellbeing through being able to provide peer support in VCSE organisations was highlighted.

**Table 1:** Demographic characteristics of participants

ID	Age	Gender	Ethnicity	Employment	Location	IMD decile*
P1	46–55	Male	White and Black Caribbean	Unemployed	Rotherham	1
P2	26–35	Female	White British	Employed	Rotherham	2
P3	26–35	Female	White British	Employed	Kirklees	2
P4	26–35	Male*	White British	Unemployed	Barnsley	2
P5	18–25	Female	White British	Employed	Leeds	2
P6	26–35	Male	African	Employed	N Yorkshire	8
P7	18–25	Female	Indian	Unemployed	Leeds	7
P8	18–25	Female	White British	Employed	Sheffield	4
P9	18–25	Female	White British	Employed	Sheffield	7
P10	56–65	Male	White British	Unemployed	York	4
P11	56–65	Female	White British	Unemployed	N Yorkshire	8
P12	> 66	Male	White British	Retired	York	8
P13	26–35	Female	White British	Prefer not to say	N Yorkshire	6
P14	36–45	Male	White British	Unemployed	Hull	7
P15	56–65	Female	White British	Employed	N Yorkshire	1
P16	56–65	Female	White British	Employed	Calderdale	1
P17	26–35	Female	White British	Employed	Doncaster	4
P18	36–45	Female	White British	Employed	Leeds	2
P19	46–55	Female	White Greek	Employed	Leeds	7
P20	26–35	Female	White British	Employed	Wakefield	8
P21	26–35	Female	White British	Employed	Sheffield	2
P22	18–25	Female	White British	University student	Sheffield	9
P23	36–45	Female	White British	Employed	Kirklees	7

**Table 2:** Summary of themes and sub-themes

Key themes	Sub-themes	Supporting quotes
Appropriate and timely support	Tailored services	*‘I was still self-harming so like at the beginning of the session she’d be like “So how was your week?” and I would be like tell her how many times I’d self-harmed and it was just felt really tailored to exactly what I needed at that time.’* *P22: White British Female, aged 18–25 years*
	Navigating complex systems	*And if your mental health problems are complex or long term it’s basically “Well we don’t deal with that” so you’re left floating in this world* *P11: White British Female, aged 56–65 years*
Fostering a sense of community	Shared experiences	*“So you go to the meetings, as many as you can, and you* *sit and listen and you identify with people and you look for* *similarities, not for differences.”* *P15: White British Female, aged 56–65 years*
	Online communities	“Since I’ve had this computer I’ve not self-harmed because I’ve had people to talk to and people just to hear me” *P1: White and Black Caribbean Male, aged 46–55 years*
Awareness of support	Knowledge of service availability	*‘I think services being well advertised would have made a big difference because I really honestly didn’t know about any services apart from just going to the GP.’* *P21: White British Female, aged 26–35 years*
	Lack of clarity	*‘you’re kind of worried then waiting for what you’re getting, you don’t know what it is, so you don’t know what to expect, like, I quite like knowing what I’m doing and what I’m expected to do and things.’* *P3: White British Female, aged 26–35 years*
Employment and education	Creating supportive environments	*“…it’s my secret, I can hide that from you, I can mask, let you know that everything’s OK and keep on doing my job, but behind it you don’t know what’s there, but had you asked the question I might have told you”* *P10: White British Male, aged 56–65 years*
	Empowerment and self esteem	*‘I do quite a lot of art that I put on my Etsy shop in my spare time as well but it’s been quite nice getting my work because of my mental health’* *P8: White British Female, aged 18–25 years*

## Discussion

In our study, participants highlighted a number of barriers to accessing both statutory and third sector organisations for support for self harm. These barriers included a perception that existing care pathways were fragmented and difficult to navigate and that services were not often suitable for individual patient needs. Participants also described how the availability of support services were not always clearly communicated. One of the perceived benefits of accessing VCSE organisations for support, was the unique sense of community or social connectedness gained in these settings. With some participants suggesting that being accepted within a community and being heard by others with similar experiences played an important role in recovery. Individuals often felt this sense of belonging was absent from primary and secondary mental health services.

The perceived social benefits of accessing VCSE support mirror findings from the recent review by Abou Seif and colleagues [10]. Both this review and the current study also demonstrated that the value of being able to provide and receive support from individuals with similar experiences was not limited to in person services, with online communities also being recognised as a source of meaningful engagement. However, the current study findings and existing literature [10] also acknowledge the potential risks associated with engaging with online content, with it being reported that some individuals may actively seek content which is likely to trigger their self-harm [13].

Participants called for greater flexibility from both statutory and VCSE organisations with regards to service access. Implementing this will require a careful balance between ensuring patient choice and customisation with protecting a feasible level of complexity within service structures; VCSE can draw on learning in statuary healthcare providers [14]. It might be also fruitful to consider a broader definition of what constitutes a provider to help provide capacity to deliver the required flexibility. Outside of the statutory and VCSE network the most visited healthcare providers are community pharmacies and there has been work exploring their potential role in supporting other services [15].

One means to achieve more connectedness and awareness of services is provided by resources that help organisations collaborate and network such as ‘sharing economies’ [16]. Cooperation can also be achieved by local authorities supporting forums for networking. For example, Tower Hamlets has such an organisation facilitated by a health lead who acts as a link between the VCSE sector and health and wellbeing boards [17]. This approach may be well placed to integrate service users and experts by experiences into resources on offer. It may also provide a means to increase resources to support volunteers, such as supervision and training.

The role education and employment settings can have in providing self-harm support was also highlighted by participants in our current study. Mental health provision in school and college settings continues to grow in the UK, with over 13,800 schools and colleges claiming grants to train new mental health leads between October 2021 to March 2023 [18]. From 2018 to 2023, there have also been 398 new Mental Health Support Teams introduced across England to help deliver mental health interventions [18]. However, there remains a lack of consensus on which type of interventions are effective in reducing mental health conditions [19]. A recent review on the effectiveness of school-based interventions to prevent self-harm, reported evidence of reduction in self-harm in 3/6 studies eligible for inclusion [20]. However, only one of these studies was deemed as methodologically sound [20]. As in the current study, recent qualitative research on facilitators to self-harm interventions in school settings, support the need for interventions to be tailored to individual needs [21]. An emphasis on the need for whole-school approaches to self-harm prevention was also noted, which is often defined by the inclusion of interventions at the whole-school level to create positive, mental health-promoting learning environments.

With regards to the provision of mental health support in the workplace, a recent review of reviews found some evidence that psychological interventions can be effective in reducing depression, anxiety and workplace stress [22]. However, there appears to be a paucity of evidence on how employment settings can effectively provide support specifically for self-harm.

Working closely with our members of our LEAG throughout the research process allowed us to develop recruitment strategy and interview procedure in which participants felt supported in sharing their experiences of non-statutory peer and community support for self-harm. This allowed us to explore a range of in-depth narratives to enable a better understanding of the importance of these services in improving public mental health and the potential barriers to accessing such support. However, despite consultation with the “Deep End” PPI panel, we acknowledge that further efforts could have been made to recruit a more diverse sample of participants, with the majority of the sample being White British, females. For example, the use of interpreters and options for in person interviews could have allowed us to further explore the voices of those which often go unheard in research on the provision of self-harm support.

Working closely with those involved in commissioning and delivering VCSE support and allowing for feedback from over 70 delegates at our stakeholder event was a key strength of the research. This allowed us to understand the implications of our results for services and the challenges of implementing service recommendations from the providers perspective.

## Conclusions

Our study suggests that VCSE organisations can play an important role in the delivery of support for people who have self-harmed. These organisations can offer participants the opportunity to receive support from individuals with shared experiences, which in turn fosters a sense of belonging that helps facilitate engagement with services. However, it is vital that appropriate supervision and training is provided to these individuals providing support to ensure their own mental health and wellbeing is protected. Further work is also required to raise awareness of the types of services available to individuals, with better communication between statutory and non-statutory services required to help individuals navigate complex care pathways, which are often viewed as inflexible and fragmented.

## Data Availability

The datasets generated and/or analysed during the current study are not publicly available due to concerns of privacy and confidentiality, but some material can be available from the corresponding author on reasonable request.

## Abbreviations

VCSE: Voluntary community and social enterprise
GP: General practitioner
LEAG: Lived experience advisory group
IMD: Indices of multiple deprivation
